# Natural Scaffolds for Renal Differentiation of Human Embryonic Stem Cells for Kidney Tissue Engineering

**DOI:** 10.1371/journal.pone.0143849

**Published:** 2015-12-08

**Authors:** Cynthia A. Batchelder, Michele L. Martinez, Alice F. Tarantal

**Affiliations:** 1 California National Primate Research Center, University of California, Davis, California, United States of America; 2 Department of Pediatrics, School of Medicine, University of California, Davis, California, United States of America; 3 Department of Cell Biology and Human Anatomy, School of Medicine, University of California, Davis, California, United States of America; UCL Institute of Child Health, UNITED KINGDOM

## Abstract

Despite the enthusiasm for bioengineering of functional renal tissues for transplantation, many obstacles remain before the potential of this technology can be realized in a clinical setting. Viable tissue engineering strategies for the kidney require identification of the necessary cell populations, efficient scaffolds, and the 3D culture conditions to develop and support the unique architecture and physiological function of this vital organ. Our studies have previously demonstrated that decellularized sections of rhesus monkey kidneys of all age groups provide a natural extracellular matrix (ECM) with sufficient structural properties with spatial and organizational influences on human embryonic stem cell (hESC) migration and differentiation. To further explore the use of decellularized natural kidney scaffolds for renal tissue engineering, pluripotent hESC were seeded in whole- or on sections of kidney ECM and cell migration and phenotype compared with the established differentiation assays for hESC. Results of qPCR and immunohistochemical analyses demonstrated upregulation of renal lineage markers when hESC were cultured in decellularized scaffolds without cytokine or growth factor stimulation, suggesting a role for the ECM in directing renal lineage differentiation. hESC were also differentiated with growth factors and compared when seeded on renal ECM or a new biologically inert polysaccharide scaffold for further maturation. Renal lineage markers were progressively upregulated over time on both scaffolds and hESC were shown to express signature genes of renal progenitor, proximal tubule, endothelial, and collecting duct populations. These findings suggest that natural scaffolds enhance expression of renal lineage markers particularly when compared to embryoid body culture. The results of these studies show the capabilities of a novel polysaccharide scaffold to aid in defining a protocol for renal progenitor differentiation from hESC, and advance the promise of tissue engineering as a source of functional kidney tissue.

## Introduction

The expanding field of tissue engineering provides hope for the creation of tissue and organs with functional properties and therapeutic potential for nearly every tissue of the human body. Initial engineering strategies have been successful, particularly for tubular structures such as blood vessels [1, 2], urinary bladder [3], larynx [4], and trachea [5]. The clinical need for functional tissue replacements is urgent for patients on the organ transplant waiting list and is particularly critical for patients waiting for a kidney; individuals in need of a kidney represent >80% of all patients on the waiting list (http://optn.transplant.hrsa.gov). It is notable that the kidney which is in greatest demand is also one of the most challenging tissues to engineer due to complex architecture, a spectrum of cell phenotypes, multiple functions, and a lack of an established stem/progenitor cell population in adults from which the kidney can be regenerated. Viable tissue engineering strategies for the kidney requires identification of necessary cell populations, suitable scaffolds to provide structural support and spatiotemporal organizational properties, as well as medium/growth factor/culture combinations to sustain growth and physiological function of the engineered tissue.

One promising approach for renal tissue engineering involves the use of natural scaffolds produced by decellularization of donor kidneys. The decellularization process typically produces a three-dimensional (3D) biological scaffold with native extracellular matrix (ECM) molecules in proper geometric locations and intact vascular conduits. Since initial proof-of-concept studies [6], decellularized kidney scaffolds have been produced in rats [7–11], nonhuman primates [12–13], pigs [14–18], and from cadaveric human kidneys failing to meet criteria for transplantation [8, 19–21]. Studies have also demonstrated the presence of other biologic agents [22] such as growth factors, cytokines [10–11, 20], and bioactive peptides that may be advantageous to growth and functional maturation of cells within the construct. Despite the promise of this approach many challenges remain including determination of a suitable source of cells for recellularization, optimization of *in vitro* culture systems for the developing tissue-engineered construct, elucidating the role and utility of the native ECM in directing cell fate, and defining an appropriate maturational endpoint prior to *in vivo* transplantation [23]. Given the shortage of available donor kidneys, tissue engineering strategies with natural scaffolding materials that are tailored to the need may further facilitate clinical translation.

Our studies have previously demonstrated that decellularized rhesus monkey kidney sections of all age groups (fetal to aged) provide a natural ECM with sufficient structural properties to support migration of cells from kidney explants in an age-dependent manner [12, 13], and the utility of these scaffolds to provide spatial and organizational influences on human embryonic stem cell (hESC) migration and differentiation [22]. To further explore the use of decellularized renal scaffolds for *ex vivo* studies of development, disease, and as engineered tissue replacements, strategies to improve recellularization were assessed. The ability of decellularized kidney scaffolds to influence cell migration and phenotype was studied with undifferentiated hESC seeded in sections of kidney versus whole kidneys. For studies on the recellularization of whole kidneys the delivery of cells through the renal artery or the ureter were assessed. In addition, to evaluate the role of the scaffold in guiding hESC renal differentiation, cells were seeded on decellularized kidney sections and compared with the cellular phenotype of cells obtained when using an organic, physiologically inert polysaccharide scaffold.

## Materials and Methods

### Tissue Collection

No animal subjects were involved in the study. A biorepository of previously obtained rhesus monkey kidney sections or whole kidneys were used for these studies; specimens were obtained through the tissue procurement program (www.cnprc.ucdavis.edu/our-services) (N = 12 included in the study).

### Scaffold Preparation

Whole kidneys or sections of kidneys were decellularized using the following protocols. Whole kidneys were perfused for decellularization as follows: (1) 100 USP/ml Heparin (Sagent Pharmaceuticals, Schaumburg, IL) in phosphate buffered saline (PBS; Life Technologies, Grand Island, NY) at 1 ml/minute for 15 minutes; (2) 1% sodium dodecyl sulfate (SDS, Life Technologies) in distilled water at 5 to 20 ml/hour (based on kidney size, e.g., ~5–10 g kidney with flow rate of 5 ml/hour for 3–4 days with a total perfusion volume of ~350–500 ml) until the tissue was transparent and the decellularization solution draining from the organ was colorless; and (3) 1x antibiotic-antimycotic (Life Technologies) in PBS wash at 1 to 20 ml/hour for 72–96 hours. Transverse kidney sections were also obtained (2–3 mm thickness) and rinsed briefly in PBS and decellularized in 1% SDS for 5–8 days at 4°C or on a continuous shaker for 48–72 hours at room temperature until translucent. SDS solution was changed daily until decellularization was completed. For decellularization at 4°C the sections and SDS solutions were brought to room temperature prior to SDS replacement. Sections of kidneys were washed at room temperature on a rotator as follows: (1) 1x antibiotic-antimycotic in PBS for 24 hours (repeated 3 times); (2) 70% ethanol for 24 hours; and (3) 1x antibiotic-antimycotic in PBS for 24 hours. Both the decellularized sections and whole kidneys were stored at 4°C in PBS with 1x antibiotic-antimycotic solution until recellularization. Prior to use, an 8 mm diameter biopsy punch (Fisher Scientific) was used for the decellularized kidney sections to ensure consistency in scaffold dimensions. Polysaccharide discs (PSS; GroCell-3D^TM^, Molecular Matrix, Inc., Davis, CA) with 8 mm diameter and 2–3 mm thickness were utilized as an organic, inert 3D polysaccharide scaffold matrix for comparison.

### Cells

All hESC studies were approved by the UC Davis Stem Cell Research Oversight Committee. Medium and reagents were purchased from Life Technologies and growth factor supplements from R&D Systems unless otherwise noted. The federally approved hESC line WA09 (H9, WiCell Research Institute) was maintained on irradiated mouse embryonic fibroblasts according to established protocols in high glucose Dulbecco’s Modified Eagle Medium (DMEM) supplemented with 20% Knockout Serum Replacer (Life Technologies), 2 mM L-glutamine, 0.1 mM nonessential amino acids, 0.1 mM β-Mercaptoethanol, and 4 ng/ml fibroblast growth factor 2 (FGF2) [24].

### Recellularization

Decellularized whole kidneys were repopulated with hESC in a closed-system custom bioreactor designed to perfuse oxygenated medium at a constant flow rate through the renal artery and/or the ureter. The scaffold was conditioned prior to cell seeding by perfusion with endothelial growth medium with supplements (EGM2, Lonza, Allendale, NJ) for at least 24 hours. hESC were prepared for seeding with the use of collagenase to remove colonies from the feeder-cell monolayer. After centrifugation to remove collagenase, hESC were dissociated to a single-cell suspension with StemPro Accutase cell dissociation reagent (Life Technologies) for 2–3 minutes. Once single cells were observed, the dissociation process was neutralized with the addition of medium. Following centrifugation, cells (20x10^6^) were resuspended in 1 ml of medium, loaded into a syringe, and infused into the scaffold via a 3-way bioreactor access port at 0.1 ml/minute flow rate. After seeding, perfusion of the construct was halted for 2 hours to allow time for cell attachment. Bioreactor culture (37°C, 5% CO_2_) and perfusion of the construct (flow rate 0.1 ml/minute) was then maintained for up to 7 days to assess cell distribution. For studies on recellularization via the ureter, tubing was used to connect the cannulated ureter to a port located outside the bioreactor and through which cells were manually infused.

Kidney sections were preconditioned for seeding by soaking in EGM2 for 24 hours then placed in a 12-well plate and gently blotted with a sterile cotton-tipped swab to remove excess medium. hESC were prepared as described above in a single-cell suspension and were resuspended in 10 μl of medium and gently pipetted onto the section-scaffold surface. Seeded scaffolds were returned to the incubator for 2 hours to allow time for cell attachment after which additional medium was added to the edges of the wells in order to ensure that the surface of the scaffold was maintained at the air-medium interface. Culture medium was changed every 3 days and kidney sections were cultured for up to 3 weeks.

### Directed Differentiation

Differentiation of hESC toward renal lineages was compared under two regimens of growth factor supplementation, denoted Protocol A [25] or Protocol B (S1 Fig) [26]. Pluripotent cells were differentiated as embryoid bodies in basal medium consisting of DMEM with fetal bovine serum (10%), penicillin (0.1 kU/ml), streptomycin (0.1 mg/ml), and L-glutamine (2 mM) in ultra-low binding plates for 24 hours prior to the addition of supplemental growth factors. Under Protocol A, cultures were supplemented with 30 ng/ml bone morphogenetic protein (BMP4) and 10 ng/ml Activin A for 2 days; then 200 ng/ml FGF9 and 1 μg/ml heparin for 5 days; followed by 200 ng/ml fibroblast growth factor 9 (FGF9), 1 μg/ml heparin, 50 ng/ml BMP7, and 1 μM Retinoic Acid (EMD Millipore) for 5 days. Under Protocol B, cultures were supplemented with 30 ng/ml BMP4 and 50 ng/ml FGF2 for 2 days; then 10 ng/ml Activin A, 100 ng/ml BMP7, and 1 μM Retinoic Acid for 3 days. At completion of the growth factor differentiation protocol, cells were plated on kidney sections or PSS as described above and cultured in basal medium at the air-medium interface. Medium was changed every 48 hours for the duration of the culture period (20 days total).

### Scaffold Analysis

Decellularized scaffolds and cell/scaffold constructs were fixed in 10% formalin for 3–6 hours and embedded in paraffin. Morphology and presence of cells in decellularized scaffolds was assessed in 5 μm sections by hematoxylin and eosin (H&E) staining. Recellularized constructs were exhaustively sectioned at 5-μm thickness with collection of 10 μm sections for molecular analysis at 10-section intervals. Every 10th section was stained by H&E to assess cell location and construct morphology, with immunohistochemistry and molecular analyses performed on adjacent sections.

### Immunohistochemistry

Analysis of immunohistochemical markers was carried out according to established protocols for glomerular, tubular, and renal developmental markers [27, 28]. The primary antibodies utilized in the study are shown in Table 1. Briefly, sections were deparaffinized and antigens exposed by heat-induced epitope retrieval in hot citrate buffer (Life Technologies). Sections were then blocked for 1 hour with a solution containing 2% normal serum of the secondary antibody host. Primary antibodies were applied and incubated overnight at 4°C. Following intermediary washes in PBS, sections were incubated for 1 hour in the dark with the appropriate secondary antibodies (Alexa Fluor 488 or 594, Life Technologies). Nuclei were visualized with the application of ProLong Gold with DAPI (Life Technologies). Slides were imaged with an Olympus BX61 fluorescent microscope, equipped with an Olympus DP72 color camera.

**Table 1:** Antibodies for immunohistochemical analysis.

Marker	Target	Vendor (Catalog #)	Dilution
Aquaporin 1 (AQP1)	Proximal tubule	Santa Cruz Biotechnology (sc20810)	1:500
Aquaporin 2 (AQP2)	Ascending Loop of Henle, Collecting duct	Santa Cruz Biotechnology (sc9882)	1:100
CD31	Endothelial	Abcam (ab28364)	1:100
Calbindin (CALB)	Ascending Loop of Henle, Distal tubule, Collecting duct	EMD Millipore (PC253LEMD)	1:100
Wide-spectrum cytokeratin (CK)	Pan-epithelial	Abcam (ab9377)	1:100
E-cadherin (ECAD)	Collecting duct	Dako (M361229)	1:100
Epithelial membrane antigen (EMA)	Proximal tubule	Dako (M0613)	1:100
Paired box protein 2 (PAX2)	Intermediate mesoderm	Life Technologies (180483)	1:100
Homeobox protein SIX2 (SIX2)	Metanephric mesenchyme	Proteintech (11562-1-AP)	1:100
Smooth muscle actin (SMA)	Mesangial cells, Vascular smooth muscle	EMD Millipore (CBL171)	1:100
Synaptopodin (SYN)	Podocyte	Progen Biotechnik (61094)	1:100
Uromodulin (UMOD)	Loop of Henle	Sigma-Aldrich (1400296)	1:100
Vimentin (VIM)	Mesenchymal, Interstitial cells	Sigma-Aldrich (V6389)	1:100
Wilm’s tumor-1 (WT1)	Metanephric mesenchyme, Podocyte	Life Technologies (MA5-12481)	1:100

### Gene Expression Analysis

DNA and total RNA were extracted from formalin-fixed paraffin-embedded (FFPE) sections with the AllPrep^®^ DNA/RNA FFPE Kit (Qiagen) according to manufacturer’s instructions. Template RNA was used to prepare cDNA with random primers (Ambion) and Sensiscript Reverse Transcriptase (Qiagen). Real time PCR was carried out in 96-well plates using the 7900^®^ ABI Sequence Detection System (Applied Biosystems) and the QuantiTect^TM^ SYBR^®^ Green PCR Kit (Qiagen) according to the manufacturer’s protocols. Primers previously identified for assessment of hESC differentiation towards renal lineages were utilized [24] as markers for pluripotency (octamer binding protein 4, OCT4), posterior primitive streak (brachyury, BRY), intermediate mesoderm (odd skipped-related transcription factor one, OSR1; LIM homeobox protein 1, LIM1; PAX2), and metanephric mesenchyme (SIX2, PAX2, WT1).

## Results

### Decellularized Scaffold Production and Characterization

**Fig 1:**
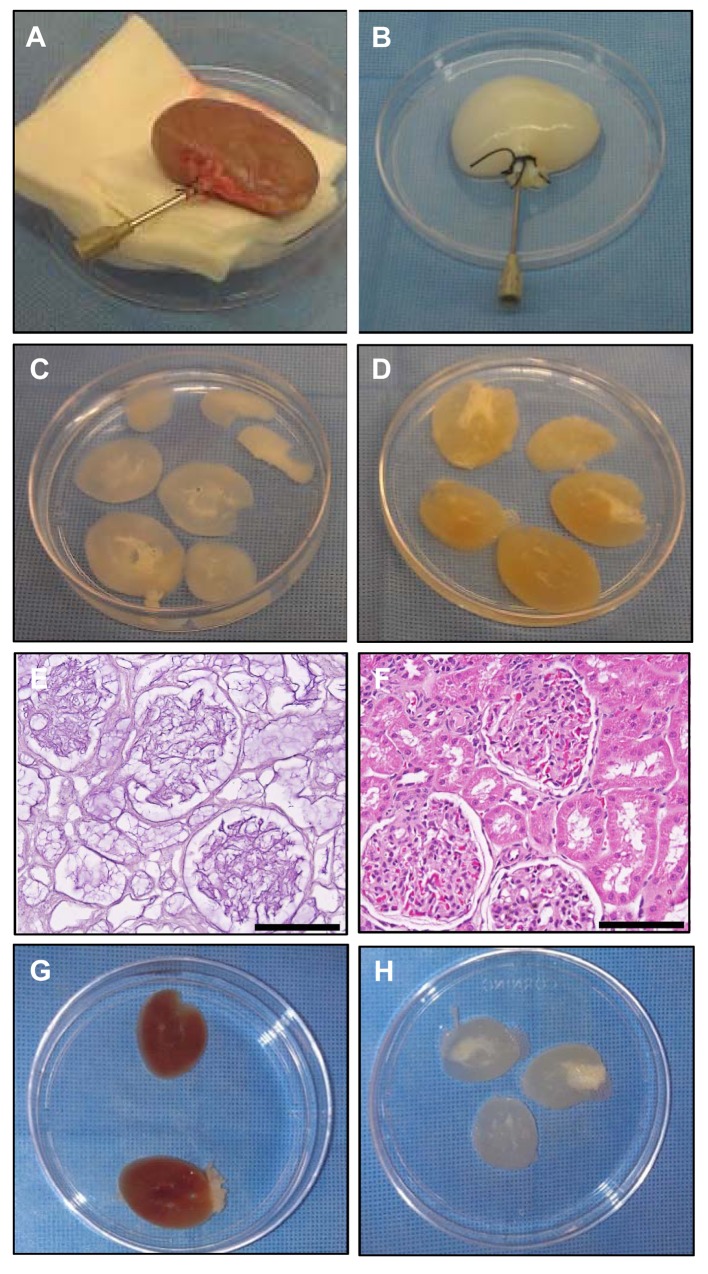
Production of decellularized renal scaffolds. Whole-kidney scaffolds before (**A**) and after (**B**) perfusion with 1% SDS. Kidneys from the same donor were compared after whole-kidney perfusion (**C**) or static section (**D**) decellularization methods. Histological comparison of decellularized whole kidney (**E**) with native kidney (**F**) demonstrated removal of cellular contents with maintenance of native renal architecture. The section decellularization process at 4°C without agitation (**G**) was greatly improved when sections were placed on a shaker during the decellularization process at room temperature (**H**). Scale bars = 100 μm.

Procedures were implemented to decellularize whole rhesus monkey kidneys by gentle perfusion of solutions in the following sequence: heparinized PBS, 1% SDS in water for decellularization, and PBS plus antibiotic-antimycotic solution to wash out residual SDS. Kidneys were opaque and transparent as the decellularization process was completed using standardized protocols (Fig 1A and 1B). Kidney sections from the same donor were compared after perfusion decellularization of the whole kidney (Fig 1C) or decellularization of sections at 4°C for 7 days (Fig 1D). Perfused kidneys were transparent while kidneys decellularized as sections retained color and opacity indicating incomplete decellularization unless they were decellularized with agitation. Histological analysis of FFPE sections of decellularized whole kidneys (Fig 1E) and comparison by H&E staining to native kidney (Fig 1F) demonstrated removal of nuclei and other cellular components with maintenance of the renal architecture. Sections of kidneys decellularized with standard protocols at 4°C or at room temperature with agitation on a shaker showed the sections prepared with agitation were fully translucent with complete decellularization apparent (Fig 1G and 1H, respectively), which was confirmed by H&E analysis. This protocol was used for all subsequent experiments.

### Scaffold Recellularization

**Fig 2:**
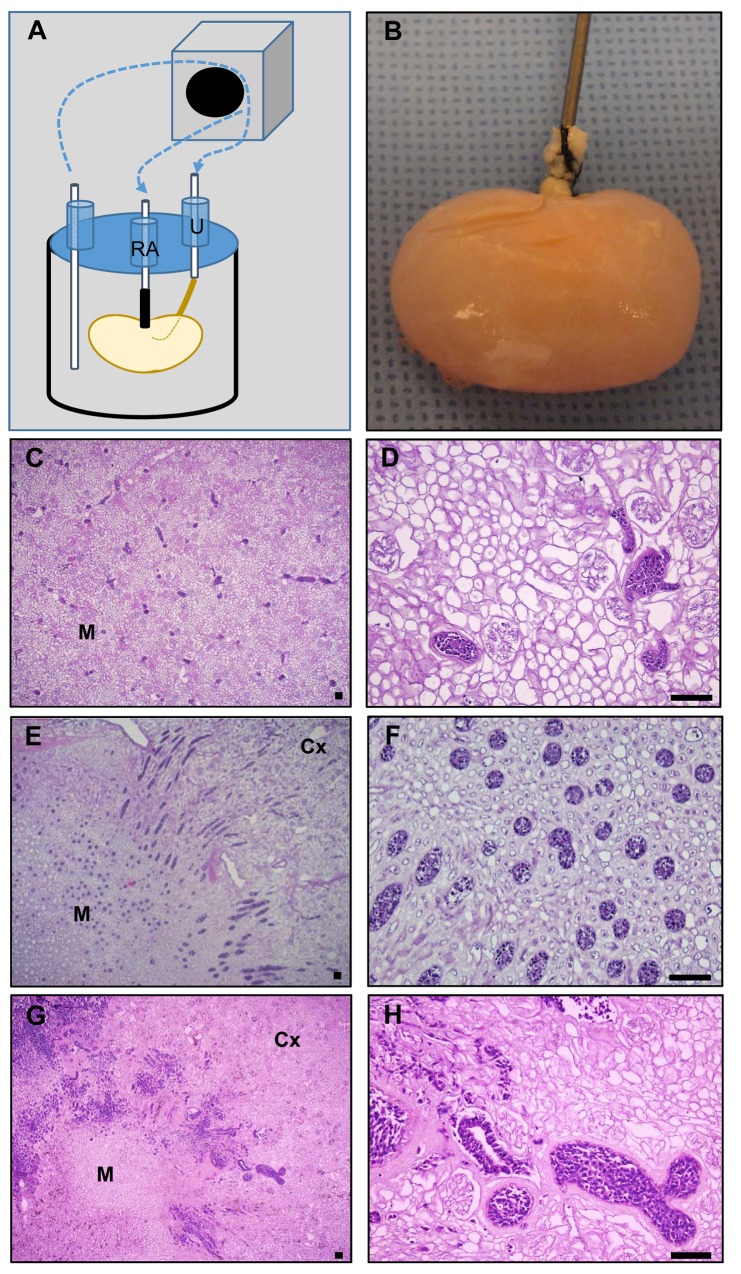
Recellularization of kidney scaffolds with hESC. (**A**) Schematic of custom-designed, perfusion bioreactor for cell seeding via the renal artery (RA) or ureter (U). (**B**) Recellularized kidneys after 7 days of culture. Cells seeded via the renal artery (**C-D**) or ureter (**E-F**) were observed in medullary vascular or tubular lumens but not in outer cortical tubules or glomeruli. With longer culture periods enhanced recellularization was observed (**G-H**), particularly in medullary regions. Representative images from a minimum of three independent experiments are shown. Medulla (M), Cortex (Cx), scale bars = 100 μm.

Studies to explore and optimize recellularization conditions for whole kidney scaffolds were conducted with a custom bioreactor designed to allow constant scaffold perfusion via the renal artery with additional ports to allow optional cell seeding via the ureter (Fig 2A). Once seeded, whole kidney constructs were maintained in culture with a visible increase in opacity over the culture period (Fig 2B). hESC administered via the renal artery (Fig 2C and 2D) or ureter (Fig 2E and 2F) were dispersed throughout the vascular or tubular spaces through which they were administered with short culture periods (<5 days). To enhance recellularization, hESC were seeded with additional growth factors including FGF, Activin A, and BMP7 followed by bioreactor culture for 7 days. Analysis of construct morphology by H&E staining demonstrated enhanced recellularization (Fig 2G and 2H). Cells typically filled vascular lumens with some evidence of epithelial morphology in tubule lumens. Cells were noted primarily in the renal pyramids and medullary rays with few cells observed in outer cortical tubules or glomeruli.

### Differentiation of hESC

**Fig 3:**
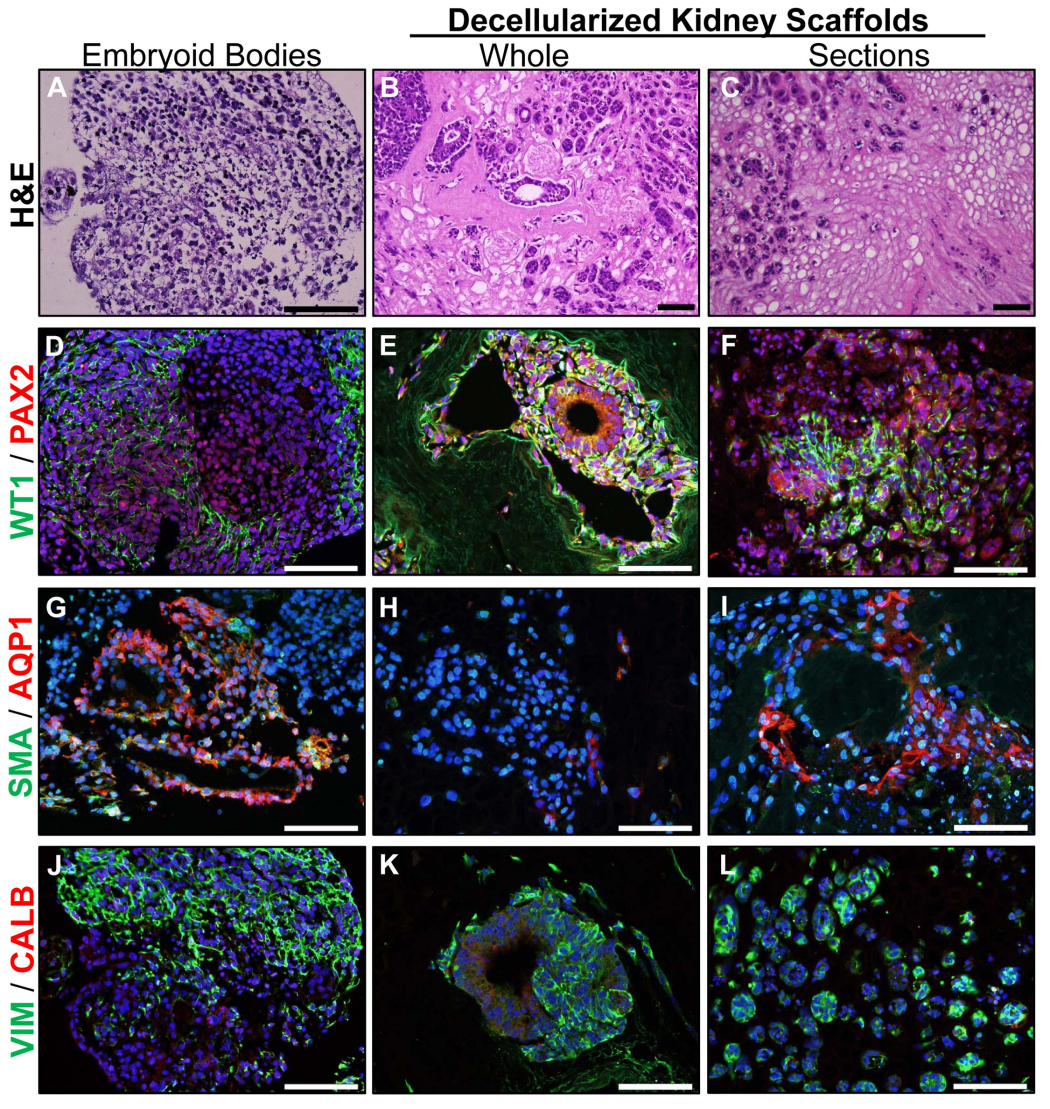
Renal developmental markers are upregulated by renal ECM. hESC were differentiated as embryoid bodies (**A**) or cultured in whole kidneys (**B**) or sections of kidneys (**C**) where cells were typically observed in the medulla and medullary rays. (**D-F**) Renal developmental markers WT1 and PAX2 were upregulated in whole or sections of decellularized kidneys when compared with embryoid body differentiation. (**G-I**) AQP1, a marker of proximal tubules, was expressed in tubule-like structures in embryoid bodies and kidney sections, but not in whole kidneys. (**J-L**) Vimentin, a mesenchymal and mesangial marker, was expressed under all culture conditions. Other markers of mature renal cell types including SMA (mesangial and vascular smooth muscle marker) and Calbindin (renal distal tubules) were not expressed. Nuclei were visualized with DAPI (blue); scale bars = 100 μm.

Whole kidneys and sections of kidney ECM were compared to assess hESC attachment, migration, proliferation, and differentiation. Additional cells were plated in suspension culture (Fig 3A) as embryoid bodies to evaluate and compare effects of the renal ECM on hESC differentiation. As noted in prior studies, hESC were found in renal papillae and medullary rays (Fig 3B and 3C) and were rarely observed in outer cortical tubules or glomeruli. Renal developmental markers WT1 and PAX2 were expressed with greater frequency on renal ECM (whole kidneys or sections) when compared to embryoid bodies (Fig 3D–3F). The renal tubule marker AQP1 was observed on cells in tubule-like structures in embryoid bodies and kidney sections (Fig 3G–3I) but rarely noted in whole kidneys. Occasional cells expressed the mesangial and vascular smooth muscle marker, SMA. Vimentin, a mesenchymal and mesangial marker, was frequently expressed (Fig 3J–3L). Calbindin, a marker of renal distal tubules, was not expressed under these conditions.

**Fig 4:**
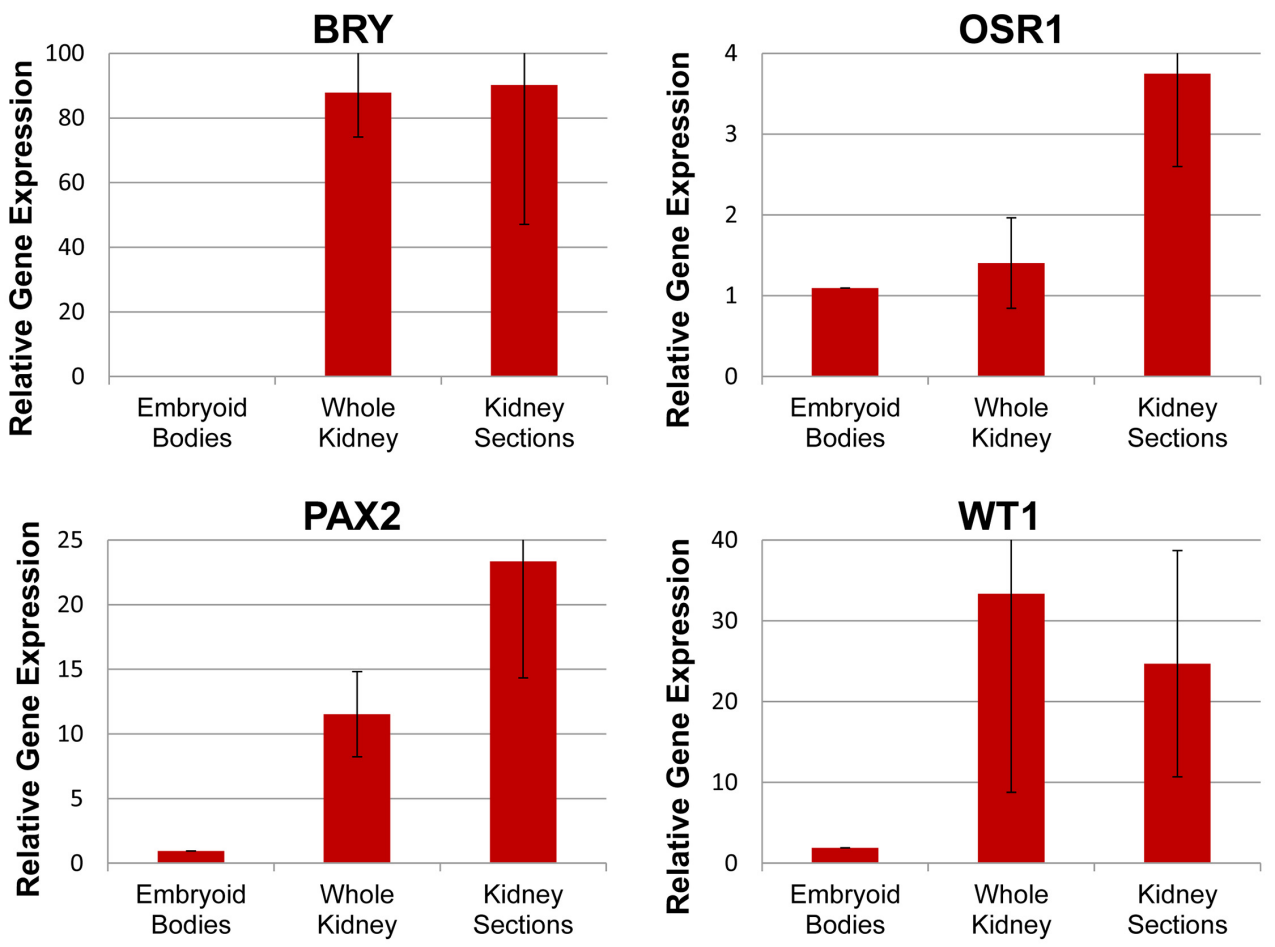
Early renal lineage genes were upregulated by renal ECM. Relative expression of mesodermal (BRY), posterior primitive streak (OSR1), intermediate mesodermal (PAX2), and metanephric mesenchymal (WT1) genes in hESC differentiated as embryoid bodies or in renal scaffolds. Mean ± standard error of the mean (SEM); N≥3 replicates.

Gene expression was compared by qPCR to assess upregulation of mesodermal and renal lineage markers. Relative expression of the mesodermal gene BRY was upregulated more than 80-fold in all constructs (whole and kidney sections) when compared to embryoid body cultures (Fig 4). Markers of intermediate mesoderm OSR1 and PAX2 were increased in kidney sections (3.7 and 23.3-fold, respectively) and PAX2 was also upregulated in whole kidneys (11.5-fold). WT1, a metanephric mesenchymal marker, was upregulated under both conditions (whole kidneys: 33.4-fold; kidney sections: 24.7-fold).

**Fig 5:**
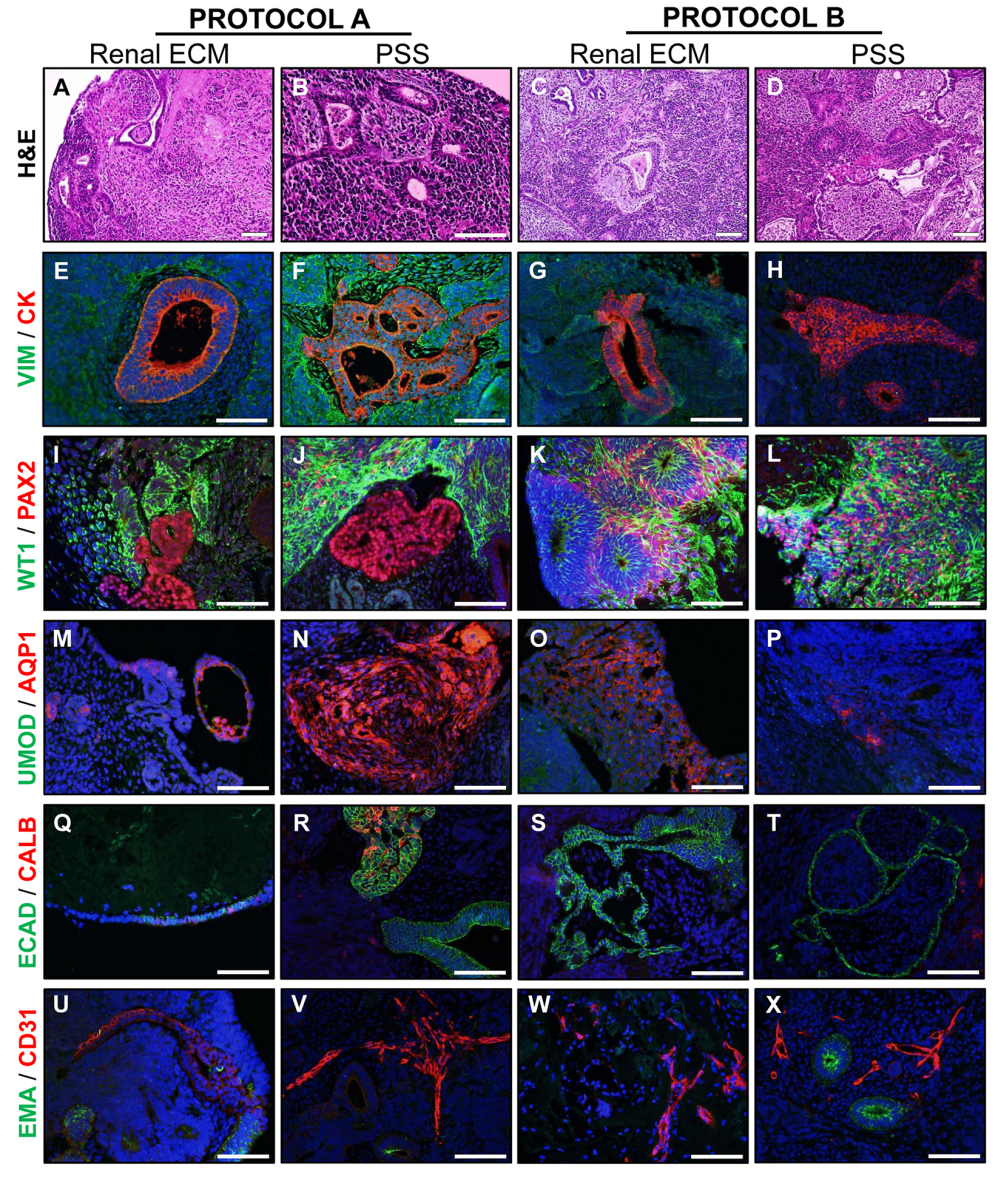
Renal-directed differentiation of hESC on renal ECM or PSS. **(A-D).** H&E staining of cell-scaffold constructs after 20 days in culture with two growth factor protocols (A or B). (**E-H**). Tubule-like structures were present and positive for CK but not VIM. (**I-L).** Regions positive for PAX2 (intermediate mesoderm and induced metanephric mesenchyme) were typically distinct from regions positive for WT1 (induced mesenchyme) under Protocol A. These markers were expressed in a more diffuse pattern throughout the construct under Protocol B. Tubule-like structures in Protocol B with renal ECM were WT1-positive and surrounded by a ring of PAX2-positive cells. (**M-P**). The proximal tubule marker AQP1 was expressed with greater frequency under Protocol A and on PSS while the Loop of Henle marker UMOD was rarely expressed in any construct. (**Q-T**). The distal tubule/collecting duct marker ECAD was widely expressed on cells with epithelial morphology in all constructs while the ascending Loop of Henle and collecting duct marker CALB was only expressed in constructs under Protocol A with PSS. (**U-X**). The proximal tubule marker EMA was expressed on some tubule structures in all constructs with small vessel-like structures positive for the endothelial marker CD31. Nuclei were visualized with DAPI (blue). Representative images shown; N≥3 experiments. Scale bar = 100 μm.

To further explore the role of renal ECM in directing differentiation of hESC, cells were differentiated towards a renal precursor fate in suspension culture with two growth factor protocols (A or B) then seeded on kidney sections at the air-medium interface for further maturation. Cell phenotype and gene expression patterns were compared with cells cultured on the natural, biologically inert, PSS. hESC differentiated into tubule-like structures of varying sizes under all culture conditions (Fig 5A–5D). Cells lining the lumen of larger tubules typically displayed an epithelial morphology and expressed cytokeratin, but not vimentin (Fig 5E–5H). Early renal lineage markers WT1 and PAX2 were expressed in distinct regions under Protocol A on both types of scaffold materials (Fig 5I and 5J) in a pattern similar to mid- to late-first trimester kidney development where the PAX2-positive ureteric bud is surrounded by WT1-positive metanephric mesenchyme [28]. Under Protocol B, PAX2-positive cells surrounded WT1-positive tubules for cultures on renal ECM with a more diffuse staining pattern observed in PSS (Fig 5K and 5L). AQP1, a marker of proximal tubules, was observed on some tubule-like structures (Fig 5M–5P) and was more frequently observed on scaffolds under Protocol A. Although weak UMOD-positive tubules were occasionally observed, this Loop of Henle marker was not present in most constructs. Markers of distal tubules (CALB) and collecting ducts (ECAD, CALB) were observed in constructs cultured on PSS under Protocol A; remaining constructs were only positive for ECAD (Fig 5Q–5T). Small vessel-like structures expressing the endothelial marker CD31 were observed in all culture conditions, as were larger tubular structures expressing the proximal tubule marker EMA (Fig 5U–5X).

**Fig 6:**
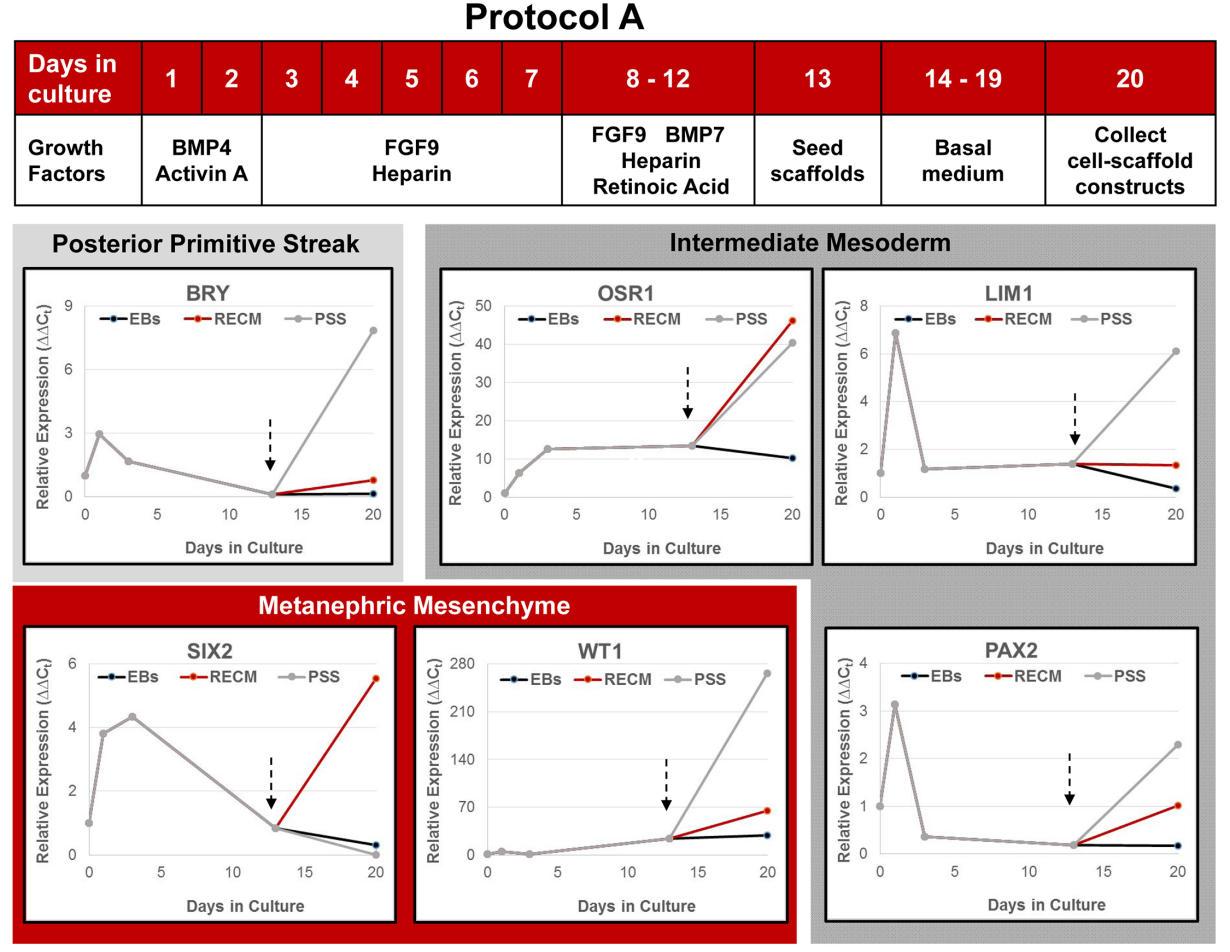
Expression of early renal lineage markers in differentiating hESC cultured under Protocol A. Pluripotent hESC were plated in suspension cultures to form embryoid bodies with supplemental growth factors as shown. Gene expression (qPCR) relative to the housekeeping gene EF1α was calculated using undifferentiated day 0 cells as the comparator. After 12 days of suspension culture, embryoid bodies were collected and plated on renal ECM or PSS at the air-medium interface in basal medium (arrows). Both scaffolds were equal or superior to embryoid body culture in upregulating renal lineage genes (N≥3 replicates). With the exception of SIX2, PSS were equal (OSR1) or superior (BRACHY, LIM1, WT1, PAX2) to renal ECM in supporting renal precursor populations.

**Fig 7:**
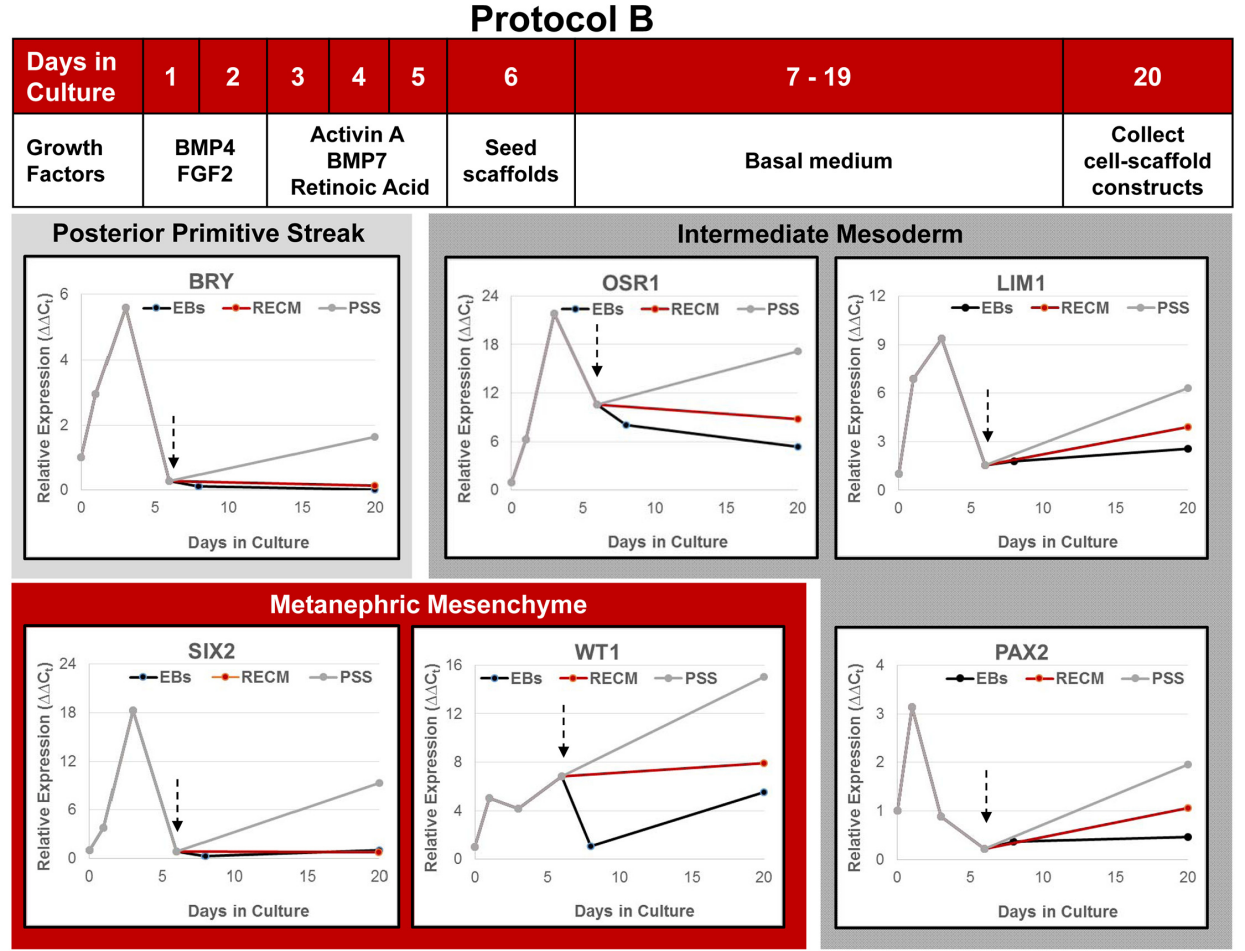
Expression of early renal lineage markers in differentiating hESC cultured under Protocol B. Pluripotent hESC were differentiated in suspension cultures as embryoid bodies with supplemental growth factors as shown. After 5 days, cells were plated on renal ECM or PSS at the air-medium interface in basal medium (arrows). Gene expression (qPCR) relative to the housekeeping gene EF1α was calculated using undifferentiated day 0 cells as the comparator (N≥3 replicates). Both types of scaffolds were superior to embryoid body cultures in directing upregulation of intermediate mesoderm (OSR1, LIM1, PAX2) and the metanephric mesenchyme gene, WT1. Renal ECM was similar to embryoid body culture in expression of BRY and SIX2, while these genes were upregulated on PSS.

Differentiating cultures and cell-scaffold constructs were assessed at multiple time points by qPCR to determine expression of renal lineage genes. Undifferentiated hESC placed into suspension culture conditions with growth factors showed a rapid upregulation of posterior primitive streak (BRY), intermediate mesoderm (OSR1, LIM1, PAX2), and metanephric mesenchyme (SIX2, WT1) genes under both Protocols A (Fig 6) and B (Fig 7). As anticipated, there was a concurrent loss of expression of pluripotency markers OCT4 and Nanog (not shown). When compared, cells differentiated under Protocol B showed a greater response across all genes ranging from 2-fold increase in OSR1 expression to 8-fold increase in LIM1 expression. The peak response under both protocols was observed on day 3 of culture for most genes with expression declining thereafter. Differentiating hESC were seeded onto scaffolds after 12 (Protocol A) or 6 (Protocol B) days of suspension culture. As a general observation across protocols, hESC responded to culture on both renal ECM and PSS at the air-liquid interface with further upregulation of the renal lineage genes, a response that was typically greater on PSS than renal ECM.

Markers of mature kidney tubule cells including CLCN5 (proximal tubule), SLC12A1 (ascending Loop of Henle), and AQP2 (collecting duct) were also assessed by qPCR. Constructs were consistently low positive for CLCN5 except for Protocol A on PSS where expression was upregulated 4-fold. SLC12A1 was not expressed, paralleling the results observed for ascending Loop of Henle marker UMOD in the immunohistochemical analysis. AQP2 was strongly upregulated in all constructs (S2 Fig), and was consistently greater under Protocol A than B (1,069-fold vs. 34-fold upregulation, respectively), and to a greater extent on PSS than renal ECM (1,050-fold vs. 55-fold upregulation, respectively).

## Discussion

Many obstacles remain before bioengineering of functional renal tissues for human transplantation can be realized. Primary hurdles include selection of suitable scaffolds to support development and maintenance of the multitude of cell types in the kidney, and the identification of appropriate stem and/or progenitor cell populations with which to recellularize and ensure both structural and functional capacity that is needed. These studies were conducted to explore the use of biologic scaffolds such as renal ECM and to compare with physiologically inert PSS that does not provide biochemical cues or the structural features of the native ECM template. Results have also refined decellularization conditions for rhesus monkey kidneys, and demonstrated that the scaffolds improve upregulation of early renal lineage markers compared with embryoid body differentiation protocols.

Recellularization was consistent in renal ECM with cells typically identified in medullary tubules or rays with little evidence of migration into the kidney parenchyma. The perfusion technology used in these studies enabled distribution of cells into all kidney quadrants in a relatively uniform manner but did not achieve the full repopulation of the endothelium or parenchymal compartments. Whether this is due to incomplete infiltration of cells to the cortical regions or the need for signals to guide the cells to migrate and attach is currently unclear. However, since kidney sections resulted in a recellularization pattern comparable to whole kidneys, incomplete perfusion/infiltration appears unlikely. Although the ECM was previously considered an inert scaffold functioning solely in structural support, many studies have since confirmed the dynamic properties of these matrix molecules with corresponding roles in tissue development, function, and repair [29]. Stem/progenitor cells are uniquely impacted by the ECM which provides mechanical and biochemical cues that regulate cell fate and thereby influence self-renewal or differentiation options [30]. How well the decellularized ECM retains these critical functions is likely to be influenced by the decellularization protocol [31], the age and health of the donor tissue [12–13], and this may vary across the many tubular and peritubular compartments of the kidney. Conversely, cells for reseeding the scaffold may be presented with varying ECM properties depending on the route of cell administration (renal artery vs. ureter perfusion; or medullary vs. cortical injection) that will likely influence migration, differentiation, and functional outcomes [32]. Further studies to examine the unique ECM configuration associated with the various compartments of the kidney will be necessary to inform further tissue engineering strategies.

The complexity of the kidney and the intricacies of the vascular and collecting systems are factors that support the use of whole-organ scaffolds with intact arteries, veins, and ureters to enable perfusion of the construct and future transplantation. Rat kidney ECM scaffolds have been recellularized with mouse ESC [6, 9], hESC [8], and human induced pluripotent stem cells (iPSC) [11] demonstrating proof-of-principle. Porcine kidney ECM scaffolds were used to enhance endothelialization of vascular structures [18], and human kidney ECM [33] using cells from amniotic fluid and demonstrated cell-scaffold interactions that aid in repopulation. The studies described herein used a proven preclinical model which provides essential insights into human health and disease [34–37]. Similarities between human and nonhuman primates include nephrogenesis, which begins in these species in the first trimester and continues throughout the mid-3^rd^ trimester, whereas rodents such as the mouse begin in mid-gestation and conclude in the postnatal period. Monkeys and humans share many characteristic features because of their close phylogenetic relationship, which aids in overcoming the roadblocks to clinical translation. While other animal models provide important insights, the rhesus monkey serves a crucial role in accelerating the clinical development of promising new therapies. For example, in order to better understand the pathogenesis of kidney disease during ontogeny, a rhesus monkey model of obstructive renal dysplasia was studied [38] and showed all of the well-characterized histopathological features observed in humans [38–39].

Biologically inert, organic, and biodegradable scaffolds such as those developed from PSS that were utilized in this study provide a unique strategy to explore the role of ECM in tissue engineering applications. These natural scaffolds provide a 3D substrate for cell adhesion without the growth factors and signaling molecules that are known to be associated with the native ECM. Outcomes were very similar when comparing PSS and renal ECM in these studies with both types of scaffolding providing a 3D structure in which tubules developed and signature markers of renal progenitor, proximal tubule, endothelial, and collecting duct populations were expressed. These results support the conclusion that the interaction of hESC in 3D culture was more influential in driving differentiation than factors associated with the renal ECM. Despite the potential of kidney tissue engineering with renal ECM, a major limitation remains the availability of human donor kidneys for decellularized scaffold production and the inherent biological variability associated with these tissues. Studies herein suggest PSS can serve as an alternative method for providing basic structural support for self-organization and differentiation of hESC toward renal lineages. Translation of tissue engineering strategies will require development of an optimized protocol for combining cells and scaffolds with the necessary culture conditions that result in consistent and highly reproducible outcomes.

Several groups have explored the use of human fetal kidneys to obtain renal progenitors [40–43] but consensus has not been established and availability will limit future translation of such a cell population for tissue-engineering purposes. The existence of renal stem/progenitor cells in the adult kidney remains unclear [44–49] and will also have limited potential for regenerating kidneys damaged by disease. In contrast, strategies to direct the differentiation of human pluripotent stem cells (ESC or iPSC) toward renal lineages have shown measures of success in the generation of early renal progenitors [25–27, 50, 51] capable of self-organizing renal structures. Landmark studies have demonstrated the potential of human pluripotent cells to differentiate into embryonic populations (ureteric bud and metanephric mesenchyme) essential for kidney formation, and cells were able to self-organize into nephron-like organoids [25]. Results of studies by Xia et al. [26] and Taguchi et al. [46] also demonstrated the nephrogenic and self-organizational properties of human pluripotent cells differentiated toward renal lineages.

Although many common growth factors were utilized across a number of published studies, a consensus protocol for renal differentiation of human pluripotent cells has yet to be established. Despite differences in timing and content of growth factor supplementation, culture at the air-liquid interface was a common feature of all successful protocols suggesting a role for oxygen tension in the renal differentiation process. Cultures developed at the air-liquid interface on filters, while showing promise in renogenic potential, remain largely 2D in nature and could be limited in size in terms of generation of functional tissue replacements. Studies with 3D cultures such as the organoid system described by Xinaris et al. [52] or the 3D scaffold system described herein will be useful for scale-up of differentiation protocols with inclusion of air-liquid interface features.

The protocols described in these studies showed that hESC could be directed to express signature genes for a number of crucial phenotypes including renal progenitor, proximal tubule, endothelial, and collecting duct populations as shown by qPCR and immunohistochemistry. Future studies to define differentiation protocols, optimal scaffolds, and 3D culture conditions will be essential to advance the promise of tissue engineering as a source of functional tissue for diseased or damaged kidneys.

## Supporting Information

S1 Fig Experimental protocols for renal differentiation of hESC. (TIF)

S2 Fig Expression of aquaporin 2 (AQP2) in differentiating hESC. AQP2, a Loop of Henle and collecting duct marker, was strongly upregulated under both differentiation protocols.
(TIF)
