# Supplementary figures and images for: Natural Scaffolds for Renal Differentiation of Human Embryonic Stem Cells for Kidney Tissue Engineering
Source: PLoS One. 2015 Dec 8;10(12):e0143849. doi: 10.1371/journal.pone.0143849 (PMC4672934; doi:10.1371/journal.pone.0143849)

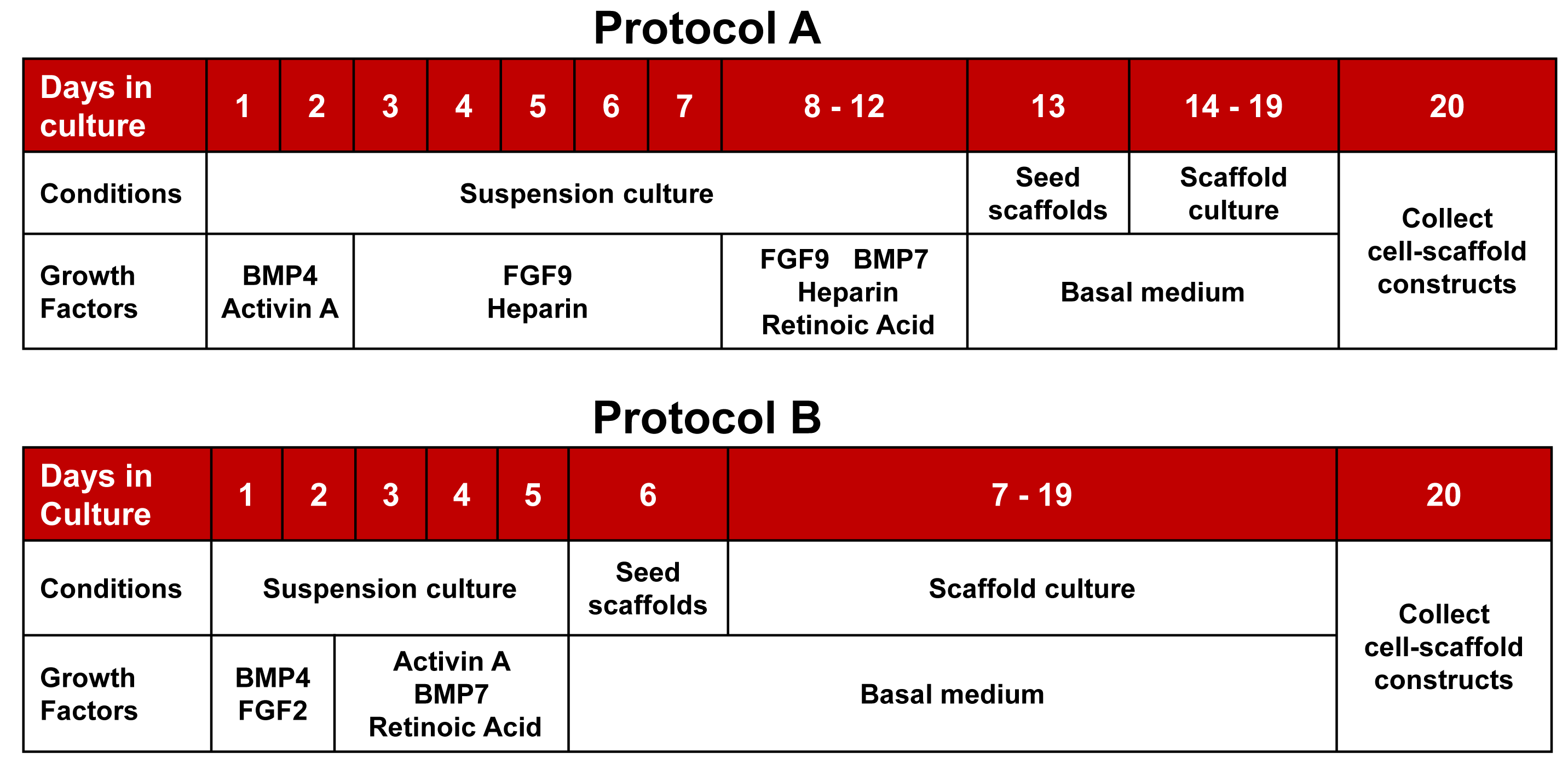

Supplement: S1 Fig — (TIF) [file pone.0143849.s001.tif]

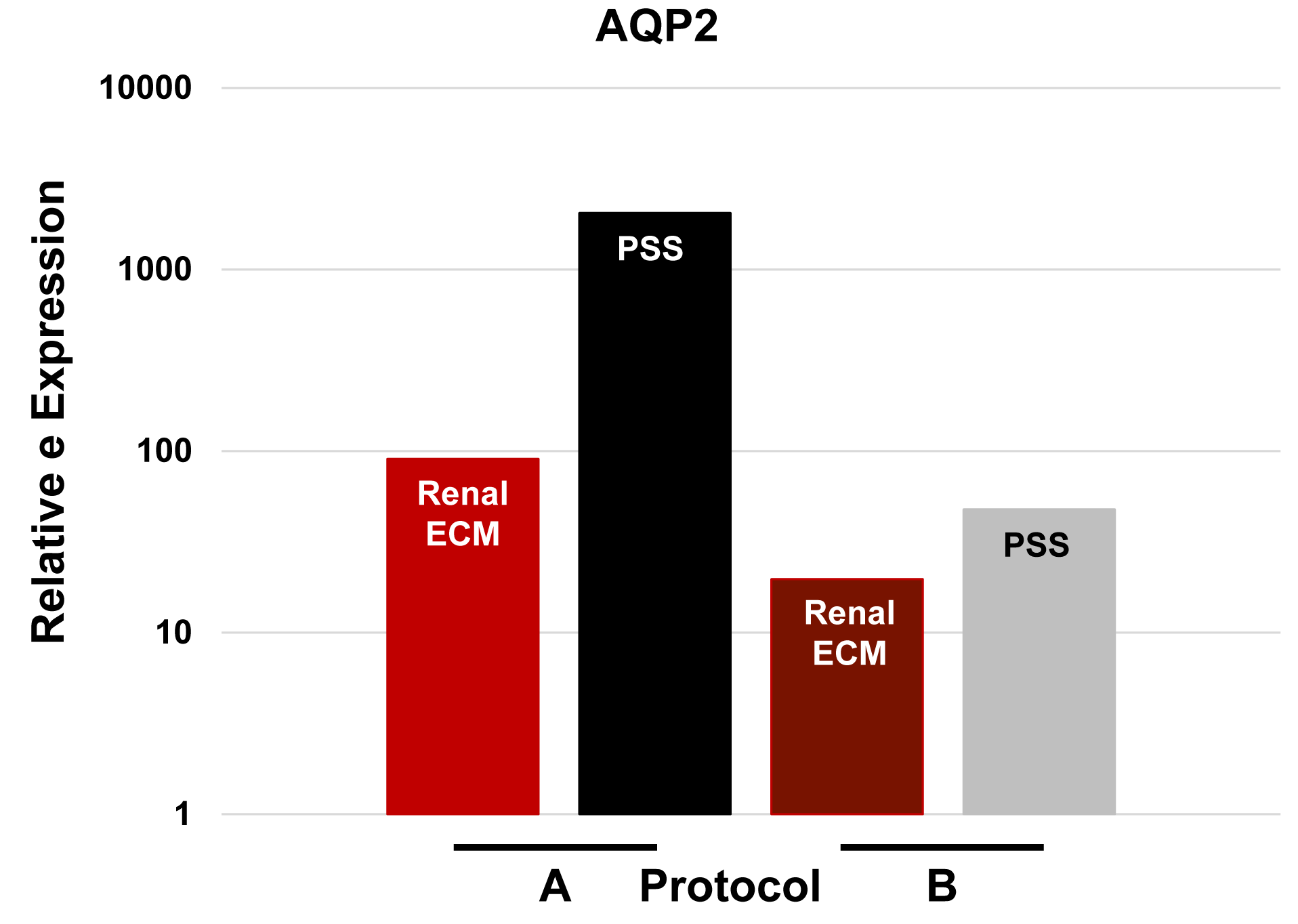

Supplement: S2 Fig — AQP2, a Loop of Henle and collecting duct marker, was strongly upregulated under both differentiation protocols. (TIF) [file pone.0143849.s002.tif]
